# Strategies to Improve Physical Activity and Nutrition Behaviours in Children and Adolescents: A Review

**DOI:** 10.3390/nu15153370

**Published:** 2023-07-28

**Authors:** Sisitha Jayasinghe, Andrew P. Hills

**Affiliations:** College of Health and Medicine, University of Tasmania, Hobart, TAS 7005, Australia; sisitha.jayasinghe@utas.edu.au

**Keywords:** inactivity, obesity, children, youth, growing years

## Abstract

Despite widespread acknowledgement of the multifarious health benefits of physical activity (PA), including prevention and control of obesity, an overwhelming majority of children and adolescents are not sufficiently active to realise such benefits. Concurrently, young people are significantly impacted by the rapid global rise of sedentarism, and suboptimal dietary patterns during key phases of development. Regrettably, the cumulative effects of unhealthy behaviours during the growing years predisposes young people to the early stages of several chronic conditions, including obesity. Clear and consistent approaches are urgently needed to improve eating and activity behaviours of children and adolescents. Based on existing evidence of “best bets” to prevent and control obesity and its comorbidities, we present a set of non-negotiable strategies as a ‘road map’ to achieving prevention and improving the health of children and adolescents.

## 1. Introduction

The health benefits of habitual physical activity (PA) are multifarious and widely acknowledged, yet contemporary trends indicate that an overwhelming majority of children and adolescents are not sufficiently active to realise these health benefits [1,2,3]. Concurrently, children and adolescents are significantly impacted by the rapid global rise of sedentarism [4]. Only an estimated 20% of young people worldwide are attaining the World Health Organization (WHO) recommended minimum of 60 min of moderate-to-vigorous physical activity (MVPA) per day [2]. Across the growing years there is typically a significant reduction in PA, with girls experiencing a more substantial decline [3]. 

Regrettably, many children and adolescents also have suboptimal dietary patterns, often consuming energy-dense, nutrient-poor foods [5], this includes adolescent girls in low-to-middle-income countries (LMICs) [6]. The cumulative effects of these unhealthy behaviours contribute to chronic conditions during the growing years such as obesity [7]. Greater effort is needed to foster positive PA and nutrition habits and establish a foundation for better health in subsequent stages [8,9].

The global prevalence of childhood obesity has reached unprecedented levels in the past three decades, and a wide range of contributing factors have been identified [10]. However, engagement in regular and developmentally appropriate PA, along with heathy dietary choices, despite being one of the most practicable obesity prevention measures, are not heeded. Young people who are more physically active typically maintain a healthier body composition than those who are inactive [11].

In this instance, an opinion piece written in a narrative style was conducted to succinctly summarize and synthesize information from various sources. These included mixed-methods evaluations, randomized controlled trials, non-randomized controlled trials, qualitative or case studies, cross-sectional studies, natural experiments, and prospective cohort studies. Unlike a systematic or structured approach to literature searching, this review did not follow a discernible method. Instead, relevant information was gathered from diverse touchpoints and thoughtfully incorporated into the ‘road map’ to emphasize the indispensable steps for enhancing PA and nutrition behaviours in children and adolescents. Furthermore, the authors’ insights, analysis, and opinions on the various concepts derived from the selected sources were utilized to present a comprehensive overview of the subject matter.

**Figure 1:**
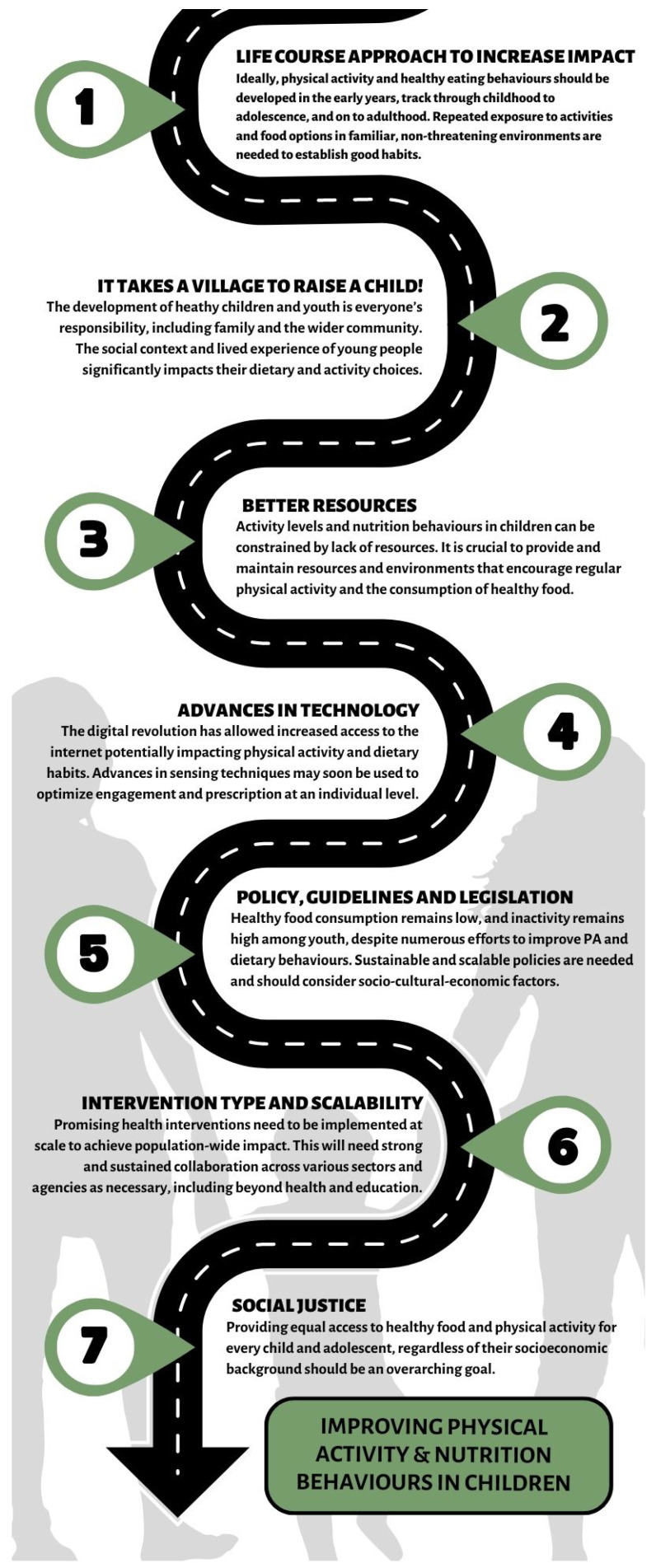
Road map of strategies to improve physical activity and nutrition behaviours in youth.

In this review, we assessed the suite of ‘best bet’ approaches to the prevention and control of obesity. It is necessary that all environments related to the growth and development of childhood make the greatest effort, including family, friends, educational environments [12] from the preschool stage, etc. [13], encouraging not only a healthy interaction with games/sports, but also with food [14]. It is essential to constantly support a wide range of physical and recreational activities [15,16]. To successfully carry out these improvements, new approaches are required not only to synthesize the available evidence [17], but to create new tools. Therefore, we merged a set of strategies to create a ‘road map’ to improve not only PA interventions, but also nutrition (Figure 1) in order to prevent and control obesity.

### 1.1. Use a Life Course Approach to Increase Impact

The use of a life course approach is valuable given that many chronic conditions, including obesity, have their genesis early in life. Traditionally used in the wider context of non-communicable diseases (NCDs) [18], understanding eating and activity behaviours during early life stages is critical to appreciate how best to intervene [19]. A poor diet during critical developmental periods can also significantly impact health and functionality [20]. 

Ideally, PA behaviours should be developed in the early years, track through childhood to adolescence, and on to adulthood. Consistent with this notion, there has been global support for the establishment of healthy PA habits from a young age, including from the WHO [21]. PA opportunities should be consistent for all children and developmentally appropriate rather than waiting until later in childhood or early adolescence, which may be far too late [22]. As for PA, the development of healthy eating habits from a young age is more likely to persist from infancy to adulthood and may have a lasting impact on one’s health [23]. 

Sadly, and despite an abundance of evidence regarding the importance of PA for children and adolescents, the very low levels of engagement suggest that the ‘advice’ is not being heeded. To establish PA habits, we must provide young people with repeated exposure to activities in familiar, non-threatening environments, including the family home and school [24]. Activity opportunities must also be nested in feasible goals and positive feedback [25], along with modification to the food environment to help promote and maintain healthy habits. This might include increasing the availability of fruit and vegetables in the home [26,27]. 

Infancy is the ideal period for all young children to develop the fundamental motor skills necessary for later engagement in PA and sport with movement across the first 1000 days of life normally characterised by increasing exploration and experimentation with a wide range of movement patterns [28]. Along with maximising motor development, infants and toddlers involved in active play also benefit from improved bone health and social connections [29], even better when parents and caregivers are role models by being physically active themselves [30]. The first 5 years (2000 days) is also crucial for the development of sound nutritional habits [31].

In a nutshell, habitual PA and healthy eating behaviours should be life-long commitments. As such, solutions are needed across the lifespan to address key drivers at multiple touch points. 

### 1.2. It Takes a Village to Raise a Child!

Family and wider community settings are significant players in the development of healthy children and youth with a child’s development collectively impacted by proximal (e.g., parents, peers, community) and distal (e.g., cultural norms, laws, customs) influences [32]. As such, an appreciation of a child’s broader social environment alongside their lived experiences directly affects their dietary and activity patterns. Young children have limited control over their food choices; however, as they mature, teachers, peers, and the media, along with parents and caregivers, may be influential. The collective community has a critical role to play in fostering behaviour patterns and influence longer-term trajectories of health behaviours. 

Parents and caregivers are crucial in determining the frequency, intensity, and modality of youth participation in PA, and are arguably in the best position to minimize the negative effects of sedentarism [33]. If we do not capitalize on childhood and adolescence to establish the motor skills (locomotor, stability, manipulative, etc.), it should be no surprise that many young people do not engage in PA. Every opportunity should be provided to support young people to acquire these skills [34] through active play, along with sharing meals with family members to enhance healthy eating habits [35,36,37]. Children’s food preferences are learned through repeated exposure to foods, so parents and caregivers should consistently introduce children to a diverse range of nutritious foods [38].

Wider systemic environmental factors (policies, funding, mass media, health, social services, etc.) can also affect children’s PA behaviour; therefore, lessons can be learnt from intersectoral, whole-of-system approaches to other contemporary public health issues [39]. Community-based interventions (CBIs) that engage multiple community actors/facilitators/influencers, and utilise existing community capacity, are central to the generation of solutions. Therefore, community members should be afforded the opportunity to design relevant real world interventions using a collective framework [40]. 

In the context of children’s nutritional habits, the school food environment is a crucial consideration with young people commonly having access to food and drinks at school, including the tuckshop, cafeteria, vending machines, classroom rewards, parties, celebrations, and fundraisers [41]. Similarly, community-based nutrition programs and initiatives such as farmers’ markets, community gardens, food co-ops, and others, can also assist families access nutritious food and help encourage healthy dietary practices.

As outlined thus far, a life-course approach, if nested within a whole-of-system ‘village’ context, may greatly improve the likelihood of positive changes in PA and nutrition behaviours. 

### 1.3. Better Resources, and More of Them, Are Likely to Improve Activity and Nutrition Opportunities

Appropriate resourcing and maintenance of environments conducive to regular engagement in PA, including the built environment, and particularly schools, are essential [42]. High-quality opportunities for active recreation, play, physical education (PE), and sports should be a priority.

Sadly, too many senior teachers and administrators perceive that PE, sport, and PA negatively impact the time available for academic instruction [43], despite the contemporary literature consistently alluding to a lack of ‘trained teachers’ and other vital support (training, funding, technology, space, administrative support, etc.) necessary to effectively manage PA within school settings [44]. A major area of focus should be the upskilling of teachers to increase the collective competency to implement PA within schools.

Another significant opportunity is active transport to and from schools [45]. Walking and cycling to school is associated with a plethora of physiological and psychological benefits [46]; however, active transport is grossly inadequate in many parts of the world [47]. A concerted effort is needed to ensure the basic and higher order needs of children (accessibility, safety, convenience, comfort, enjoyment, etc.). Despite being uniquely positioned, schools are not solely responsible, with stronger school–community partnerships being an equally important goal.

Alongside PA, schools are also well placed to encourage dietary practices among students and the wider school community. Educators face various obstacles, including inadequate course materials, limited time, insufficient training, and unhealthy food close to schools [48,49,50]. Potential strategies include a cross-curricular approach to nutrition education [51,52] and collaborative approaches among government and non-government organizations responsible for promoting school health education [50,53].

### 1.4. Technology Is Here to Stay—Use Devices Wisely!

Habitual PA in all settings has drastically decreased and/or been replaced by labour-saving devices and artificial intelligence (AI). The trajectory of the digital revolution has been profound, with instant accessibility to the Internet on mobile devices, along with the potential to overindulge, negatively impacting PA levels. These vicissitudes are more evident in younger generations (e.g., millennials and Gen Z) and have contributed to surges in the prevalence of lifestyle related diseases, including obesity [7]. Novel approaches are urgently needed to minimise the negative impacts of technology on the PA levels of young people, and, instead, use technology to promote PA [54,55,56,57,58,59,60]. This may include exergaming [61] to capitalise on children’s interest in games, and motivate more children to develop motor skills [62]. Gamification—the practice and application of game elements in a non-game context—plus augmented reality could also be a viable option to increase habitual PA [63] and generate sufficient intensity to meet health recommendations in children with obesity [64]. The decision to use active gaming in schools should be based on individual school needs and goals and accompanied by efforts to promote regular PA outside of school.

Wearable technologies could also be a cost-effective way of improving habitual PA [65,66], including pedometers, sports bracelets, sports watches, and accelerometers. with a high degree of personal preference for users [67,68]. Interestingly, limited extant data indicate that wearable devices in a PA intervention can result in significant improvements in body weight and composition in children and adolescents [69]. Nevertheless, with the recent advent of activity trackers specifically designed for children (e.g., KidFit, Fitbit Ace, LeapFrog LeapBand, Sqord Activity Tracker, Garmin Vivofit Jr 2, etc.), more robust research is needed [70].

Many contemporary techniques can also collect, transfer, and interpret data instantaneously and provide more granular data about individual preferences, habits, and behaviours. In the not-too-distant future, it may be possible to optimise both engagement and prescription at an individual level using advanced sensing techniques and AI algorithms. Personalised PA, supported by technology, will likely be a key ingredient for PA enhancement in younger people [71], including the use of ‘rewards’ for the use of devices linked to the completion of defined physical activities or tasks.

The deployment and success of tech-based PA interventions in schools will depend on buy-in from school staff and their willingness to adopt novel strategies. The limited literature to date indicates that teachers acknowledge the positive aspects of technology such as increased motivation, competition, and activity levels, but are apprehensive about safety, surveillance, and cost [72], perhaps because the use of technology may be considered a double-edged sword. Increased utilisation may lead to inadvertent increases in both screentime and physical inactivity, plus alterations in sleep and potential reductions in academic performance [73]. The ‘tracking’ of data using technology may have numerous additional drawbacks, including with respect to anonymity, privacy, and consent. It is particularly important that universal guidelines are established to manage the use of such technologies with minors.

In recent times, our relationship with food has also been transformed [55], with the rise in globalisation and inequities in modern food systems making it harder for many to access safe and nutritious food [56]. At the same time, increased production of manufactured, refined, repackaged, and ultra-processed foods [57] and consumption of foods lower in nutritional value has escalated [58]. In contemporary lifestyles, urbanisation and increased parental work hours have significantly impacted the dietary habits of children [59], including the overconsumption of convenience foods [60].

As for PA promotion, there is tremendous potential to enhance nutritional outcomes by utilising mobile and wireless devices [74]. The emergence of mHealth approaches, in response to the widespread use of mobile technologies, has led to the adoption of tools such as text messages, mobile apps, sensors, wearable devices, and other wireless monitors [75,76]. Numerous approaches exist to promote better nutrition in children with video games and computer usage being very popular, including ‘advergames’ and ‘exergames’ [77]. Children’s natural inclination towards electronic media, their tendency to dislike didactic methods, and their fondness for interactive content have all contributed to the effectiveness of such approaches [78]. More recently, there has been a widespread adoption of computerized chatbots, also referred to as conversational agents, to emulate human health coaches, primarily because they can efficiently and intuitively monitor diet and energy balance. However, it is uncertain whether such chatbots can be effective at providing ‘nudges’ to improve behaviour [79,80].

### 1.5. Implementation, Realistic Evaluation and Flexibility Are Critically Important!

Despite numerous initiatives to improve PA behaviours, levels of PA remain low and inactivity in youth remains high, as it is challenging to generate sustainable policies and implementation plans that are scalable and consider and consider relevant socio-cultural-economic factors [81,82,83]. Collaboration among politicians, bureaucrats, and advocacy groups is essential for successful implementation of public policy in PA [84,85,86].

A range of school-based approaches have demonstrated varying levels of success in different settings [87], including in relation to the effectiveness of mandating PE lessons [88], promotion of active transport, increased provision of extracurricular activities, and structured recess periods [88]. If chosen wisely and implemented optimally, many policy initiatives have the potential to positively impact PA levels in children.

Public health law, which promotes the understanding, development and use of law as a tool for promoting health, is well advanced in relation to tobacco and alcohol control [89], and despite being in its infancy with respect to PA, it may be useful [90]. Fiscal incentives for being active at an individual level and taxation strategies that incentivise ‘human-powered/active transport’ have also been efficacious in multiple settings [87]. Regarding children and adolescents, mandating PE, outdoor play, walking and biking programs and free access to community sport facilities could provide substantial benefits; however, they may have unintended consequences [91]. There is also an urgent need for extensive work in testing and realistic evaluation of policies, optimising the use of scientific evidence and an enhanced knowledge of theories and models that inform PA policies [91].

Across several decades, a proliferation of ‘PA guidelines’ have been developed [92]. Despite ‘best efforts’, the variability in quality, target age groups, activity modality, prescribed duration, intensity, and frequency has highlighted the need for greater consistency in methodological approaches in the development of population-specific guidelines [93] and the use of a stronger ‘evidence-based’ approach [94]. Moving forward, a pragmatic consideration of theories of behaviour change (social cognitive, humanistic, socioecological, etc.) in the design, implementation and evaluation of policy/guidelines is necessary [95].

The landmark WHO series ‘Essential Nutrition Action’ sets out clear, prescriptive standards for developing national multisectoral action plans to reduce risk factors. This includes strategic leadership and coordination roles in different sectors, integrated policies to promote healthy growth, manage overweight and obesity and promote healthy diets, and addressing the social determinants of health and health equity [96,97]. Policy initiatives and guidelines must include stronger regulations and enforcement of food sold in and around schools [98], along with stronger rules on food advertising in the digital sphere [99]. It is imperative to implement policies that prioritize the well-being of vulnerable and marginalized communities to eradicate food insecurity and discourage the promotion of unhealthy foods [100]. By prioritising implementation, realistic evaluation, and flexibility alongside policies, guidelines, and legislation, we can better support children in developing healthy eating habits that last a lifetime.

### 1.6. Intervention Type and Scalability—What Is Ideal, What Is Possible?

Several school-based PA interventions have been reported [101,102,103], with most intervention types belonging in one of three categories: (a) the expansion of opportunities for youth to be more active on a new occasion; (b) the extension of an existing PA opportunity by increasing the amount of time allocated; and (c) the enhancement of existing PA opportunities through strategies designed to increase activity above routine practice [104]. The case for interventions to address sedentary behaviours is similar [105,106], with no consensus on the most effective intervention type/modality and the supportive behavioural/ecological model in children and adolescents. As such, mixed-methods interventions that address context-specific needs are prudent.

The type and scalability of an intervention may mean the difference between effective/sustainable or mediocre outcomes [107]. PA interventions limited to small numbers in controlled settings are of modest benefit in the context of an obesity epidemic; however, given the extent of the problem, requires greater emphasis on large-scale practical solutions that work in varied real-world settings. Some national, whole-of-school initiatives such as the ‘Comprehensive School Physical Activity Programs’ in the US, and ‘Creating Active Schools’ in the UK have shown promise; however, there is a lack of global consensus on how to effectively scale up PA interventions in schools [108].

The scaling up of PA interventions requires strong and sustained collaboration across multiple sectors and agencies, and is not limited to health and education [108]. Unfortunately, promising interventions are often stymied by lack of knowledge, skills and capacity among policymakers and practitioners, along with political impediments and resourcing limitations [109].

There is also a growing focus on incorporating nutrition-sensitive approaches into large-scale public health nutrition initiatives such as interventions in agriculture, social protection programs, health education, and behaviour change communication campaigns [110]. More information is also becoming accessible regarding the optimal methods to execute interventions on a large scale, while also concentrating on crucial periods of life such as childhood [111,112]; however, scaling up of nutrition strategies that benefit children and adolescent *en masse* is extremely resource intensive. To illustrate, a recent World Bank report highlighted that an additional USD 10.3 billion annually in public funding would be required to adequately address undernutrition on a global scale [113]. Therefore, despite improvements, there is still much work to be done.

### 1.7. Social Justice—Sport and Food Security as Tools?

As referenced in the socio-ecological model of PA, a mixture of factors are influential in determining the adequacy of PA participation [114]. Minority groups, including people of colour, aboriginal peoples, ethnic minorities, people with disabilities, women and individuals living in regional areas and/or lower SES neighbourhoods, often languish at the bottom of the social hierarchy and are more likely to have suboptimal physical (e.g., safe, affordable, and quality PA infrastructure) and societal (e.g., leisure-time, peer support, etc.) supports for being active [115]. Typically, such individuals also face significant barriers to being habitually active and commonly display systemic disparities in health outcomes related to inactivity. Despite some limitations (e.g., lack of scalability) in particular settings, sport is a viable method to assist in closing the gap of PA-related social injustices throughout the social hierarchy [116].

Engagement in sport and PA is a vital component of the toolkit to combat inactivity in children and adolescents and foster improvements in body composition. Participation in sport has the potential to improve fundamental movement skills and contribute to improvements in cardiorespiratory fitness [117,118]. In addition to the physical benefits, participation in sport can also provide educational and psychosocial benefits across the growing years [119,120]. Nonetheless, the literature is replete with barriers (e.g., reduced access to PA facilities, working parents, unsafe neighbourhoods, substandard PE curricula, etc.) that have rendered the attainment of benefits exceedingly challenging [121]. Unfortunately, access to sporting opportunities is not equitable with financial limitations postulated to be at the forefront of the widening disparity between child/adolescent sport participation rates in low and high SES regions [122]. Disturbingly, many of these inequalities are transgenerational, paving the way for the perpetuation of a cycle of disadvantage related to engagement opportunities in PA/sport [123].

Are financial incentives the answer to increased opportunities for sport in children and adolescents? Reducing structural inequality in sport requires more than just money [121], a sustainable whole-of-system approach must be adopted to allow schools, policymakers, and parents to work together to improve youth engagement in sport. Recent literature exemplifies how ‘locally owned’ community sport interventions are particularly efficacious in empowering youth and transcending several societal inequities related to PA [124]. From the point of view of children and adolescents, it is important to note that sport provides them invaluable opportunities for civic engagement, improvement of cognitive/emotional functioning and subsequently, improved physical and psychological well-being [125], along with opportunities to break down cultural barriers, build community identity, establish friendships, develop networks, and reduce social isolation [126]. It is also prudent to combine engagement in sport with appropriately designed health literacy material, the ability to seek, understand and act on health information and services [127], and form the basis of behaviour patterns that benefit health in the long run.

Social justice in relation to youth nutrition requires that every child and adolescent, irrespective of their socioeconomic background, is able to obtain healthy and nourishing food [128]. Acknowledging that food insecurity is a type of social injustice that impacts vulnerable groups, including low-income families, communities of colour, and individuals residing in food deserts, is a crucial component of this movement [129,130,131]. By guaranteeing access to healthy and nutritious food for all students, school-based nutrition programs can be instrumental in advancing social justice by prioritizing equity and inclusion during the planning and implementation phases of such programs [132]. By promoting equitable access to nutritious food and fostering a culture of health and well-being in schools, we can help to promote social justice and create a more just and equitable society.

## 2. Limitations

The narrative approach utilised in this opinion piece lacks the precision of a more structured synthesis of evidence such as a systematic or a systematised review. Furthermore, there was no formal assessment of quality of the literature included, aside from the authors’ interpretation of its merit in value adding to the conceptual arguments advanced in relation to the ‘road map’.

## 3. Strategic Perspectives

**Figure 2:**
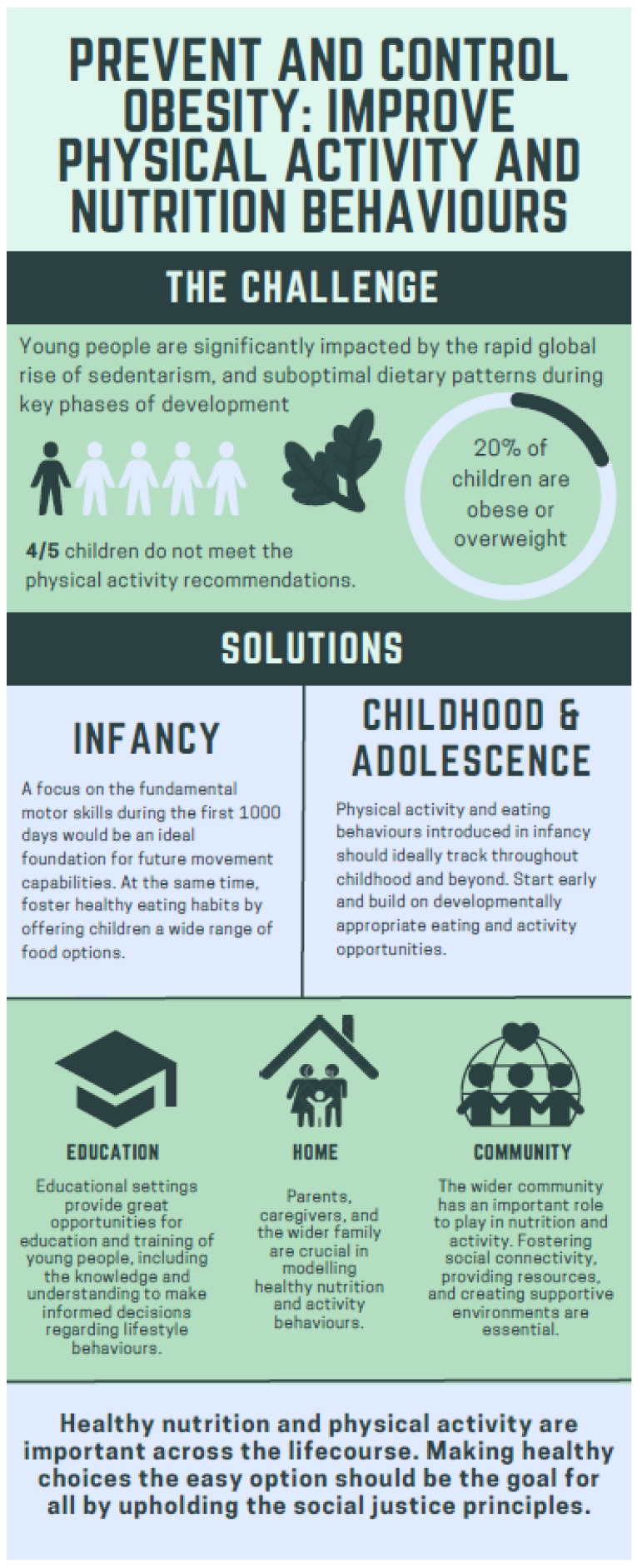
Preventing and controlling obesity.

The inactivity pandemic and poor nutrition habits in children and adolescents are complex and influenced by a range of social, environmental, and cultural factors. We contend that a roadmap to prevent and control obesity through nutrition and PA should take a life course approach, involve whole-of-system thinking, ensure adequate resourcing, optimise the use of technology existing policies, scalable interventions, and social justice, is a non-negotiable starting point (Figure 2). A logical next step is to focus on research priorities, intervention and implementation strategies pertaining to sedentarism—the co-dependent but distinct construct of physical inactivity [133]. Likewise, prioritizing the development of interventions that assist children and adolescents who are at risk of experiencing food insecurity should be a public health focus [134]. Compositional analyses in relation to time use could provide valuable insights into how children and adolescents spend their waking hours and the potential impacts on their health and well-being, including examining time spent on various PA, sedentary behaviour, and sleep, compositional analyses could help identify patterns of behaviour that may be associated with positive or negative health outcomes [135]. Compositional analyses, particularly when combined with life course epidemiology, can be vital in the development of strategies and interventions aimed at promoting healthy behaviours and addressing potential risk factors [136].

Despite global leadership from the WHO and other agencies (e.g., Global Action Plan on Physical Activity 2018–2030, WHO 2021—Promoting physical activity through schools: a toolkit, etc.), there is much to be done to advance the global PA agenda [94]. For instance, a significant body of translational work is yet to be implemented to decipher findings related to PA behaviour (active transport, sport, PE, etc.) into settings (communities, schools) by various vehicles (mass media/advertising, policy) [137]. More investment is needed in sustainability research and implementation science to unearth ways of eliciting maximum health benefits from evidence-based research in PA [138].

Worldwide, an estimated 80% of youth fail to attain the recommended minimum 60 min of MVPA per day [4]. Concurrently, evidence links physical inactivity in children and adolescents and adverse physical, mental, social, and cognitive health outcomes, including overweight and obesity. As such, improving PA levels in youth should be a much higher public health priority. The case for nutrition is similar. Although the WHO and UNICEF have provided guidance on nutrition, there is still progress to be made in safeguarding the health of our children [139], with 144 million children under 5 suffering from stunting, 47 million being wasted, and 38.3 million being overweight in 2019 [140].

## Data Availability

Not applicable.
